# Supplementary material for: SyNDI: synchronous network data integration framework
Source: BMC Bioinformatics. 2018 Nov 6;19:403. doi: 10.1186/s12859-018-2426-5 (PMC6219086; doi:10.1186/s12859-018-2426-5)
Supplement: Supplementary file 5 — Files of the example presented in Additional file 4. This additional file is a zip package that contains all files used in Additional file 4. Detailed descriptions of each of these files are presented in a README file included in this zip package. (GZ 30411 kb) [file 12859_2018_2426_MOESM5_ESM.gz › SyNDITools/Meme2Fimo/meme_4.9.0_4.tar.gz!/meme_4.9.0/doc/ama-qvalues.html]

ama-qvalues


# ama-qvalues

**Usage:** `ama-qvalues [options]`

**Description:**

Read an AMA output file and output each sequence name, p-value
and q-value. At the end of file, the value of pi\_0 (number of
sequences not showing significant binding to motif) is shown,
unless there are fewer than 100 p-values in the input.

**Input:**

Reads `standard input` expecting an AMA output file.

**Options:**

-seed <seed>
:   seed for random numbers; default: 1

-bootstraps <boot>
:   number of bootstraps to perform while computing pi\_0; default: 1000

**Output:**

For each sequence in the input, outputs the sequence name, the p-value of the sequence and the
q-value (minimum false discovery rate).

If there are more than 99 sequences with p-values in the input then it provides an estimate of
the number of sequences that contain the motif, otherwise known as the pi\_0.

**See also:**

qvalue utility
